# The Role of Rotational Thromboelastometry in Early Detection of the Hemostatic Derangements in Neonates with Systemic Candida Infection

**DOI:** 10.3390/jof11010017

**Published:** 2024-12-30

**Authors:** Rozeta Sokou, Alexia Eleftheria Palioura, Aikaterini Konstantinidi, Alexandra Lianou, Maria Lampridou, Martha Theodoraki, Daniele Piovani, Stefanos Bonovas, Konstantina A. Tsante, Petros Ioannou, Nicoletta Iacovidou, Andreas G. Tsantes

**Affiliations:** 1 Neonatal Intensive Care Unit, “Agios Panteleimon” General Hospital of Nikea, 18454 Piraeus, Greece; al.palioura@gmail.com (A.E.P.); kmaronia@gmail.com (A.K.); alexlianou95@gmail.com (A.L.); marialampridou1@hotmail.com (M.L.); anastasiosmmr@yahoo.gr (M.T.); 2 Neonatal Department, National and Kapodistrian University of Athens, Aretaieio Hospital, 11528 Athens, Greece; niciac58@gmail.com; 3 Department of Biomedical Sciences, Humanitas University, Pieve Emanuele, 20090 Milan, Italy; dpiovani@hotmail.com (D.P.); sbonovas@gmail.com (S.B.); 4 IRCCS Humanitas Research Hospital, Rozzano, 20089 Milan, Italy; 5 Laboratory of Haematology and Blood Bank Unit, “Attikon” Hospital, National and Kapodistrian University of Athens Medical School, 12462 Athens, Greece; ktsante@yahoo.com; 6 Department of Internal Medicine & Infectious Diseases, University General Hospital of Heraklion, 71110 Heraklion, Greece; 7 Microbiology Department, “Saint Savvas” Oncology Hospital, 11522 Athens, Greece

**Keywords:** neonates, invasive fungal infection, *Candida* infection, candidemia, hemostasis, coagulation status

## Abstract

Background: Systemic *Candida* infection (SCI) is the third most common cause of late-onset sepsis in Neonatal Intensive Care Units (NICU). While platelet involvement in fungal infections has been extensively studied, evaluation of the hemostatic mechanism in Candida infections, especially in neonates, has not been widely investigated. The aim of the current study was to evaluate the hemostatic profile of neonates with SCI through rotational thromboelastometry (ROTEM), a laboratory method that assesses the viscoelastic properties of blood. Methods: This is a single-centered prospective cohort study including a group of neonates with SCI (*n* = 21); the control group consisted of healthy neonates (*n* = 24). Demographics, clinical parameters, and laboratory data were recorded at the disease onset. Neonatal scores for the assessment of disease severity (Modified NEOMOD, nSOFA, and NeoBAT) were also calculated. ROTEM parameters of neonates with SCI were compared to those of healthy neonates. Results: ROTEM parameters differed between neonates with SCI and healthy neonates, indicating a hypocoagulable profile of infected neonates. Specifically, neonates with SCI had significantly prolonged clotting time (CT) and clot formation time (CFT), as well as lower clot amplitude at 10 min (A10) and maximum clot firmness (MCF) when compared to healthy neonates (*p* values < 0.05), findings that remained consistent after adjusting for confounding factors such as gestational age, birth weight, and sex. In addition, a strong correlation was noted between ROTEM parameters and disease severity based on the modified NEOMOD, nSOFA, and NeoBAT scores. Conclusions: ROTEM parameters revealed a hypocoagulable profile in neonates during the early stages of SCI, which is also associated with disease severity. The results of this study highlight the need for monitoring of hemostatic status of this vulnerable group of patients and indicate that ROTEM analysis may have a role in the early detection of the hemostatic derangements associated with SCI in neonates, in order to ensure timely diagnosis and targeted therapeutic intervention.

## 1. Introduction

*Candida* infection is one of the main causes of morbidity and mortality in the Neonatal Intensive Care Units (NICUs), especially in premature and low birth weight neonates (LBW) [1,2]. Studies report that premature and very low birth weight (VLBW) neonates are at particularly high risk, with rates of candidiasis ranging from 7% to 20% in developing countries [3,4]. LBW neonates, and especially extremely low birth weight (ELBW) neonates, often experience complications associated with invasive candidiasis, such as liver and kidney abscesses, meningitis, and endocarditis [5,6,7], situations that need prompt diagnosis and management. In 70% of cases, *Candida* species are isolated from the blood, in 15% from the urine, in 10% from the cerebrospinal fluid (CSF), and in 5% from the peritoneal fluid, with a mortality rate reaching 30% in LBW neonates [8]. Superficial infections, such as oral candidiasis and diaper dermatitis, although milder situations than systemic-invasive candidiasis, are also reported to be associated with increased mortality and negative impact on the neurodevelopmental progress of surviving neonates [9]. 

The incidence of systemic *Candida* infection (SCI) varies up to 20-fold across different healthcare centers, which is associated with compliance to preventive protocols [4,10,11]. Although timely diagnosis and immediate initiation of treatment improve outcomes, morbidity and mortality remain high. Invasive candidiasis is a relatively common cause of late-onset sepsis in NICUs, presenting with non-specific clinical symptoms and laboratory findings, delaying timely recognition and making diagnosis challenging [12]. *Candida* isolation is difficult, as cultures of blood or other sterile body fluids, “gold” standard methods for the diagnosis, have low sensitivity and they are time consuming [13,14]. When the results of cultures are available, the disease may have already spread with multi-organ involvement, and worsening of the prognosis. Regarding laboratory findings, leukocytosis or leukopenia, as well as hyperglycemia have been reported [12]. The possibility of developing SCI increases proportionally with blood glucose levels, indicating an additional marker for disease aggravation [15]. It has also been proven that thrombocytopenia is linked to a higher risk for SCI [15,16]. 

The coagulation system plays an important role in homeostasis and the body’s defense against pathogens, as it is involved in the response to harmful stimuli and the inflammatory process [17]. The human plasma contains coagulation factors that stimulate the accumulation of microorganisms such as fungal pathogens. This accumulation possibly facilitates the adhesion of pathogens on the host cells, allowing the subsequent invasion of the tissue. According to studies, such as that of Amara et al. [18], fibrinogen and other coagulation factors not only promote the adhesion of fungi but they can also activate the contact system, which is linked to the inflammatory and defense mechanisms of the host. In experimental data, it has been observed that the conidia of the Paracoccidioides brasiliensis form aggregates in the presence of human plasma, reinforcing this hypothesis [19].

While the implication of platelets in various types of fungal infections have been extensively studied [20,21], data concerning the interaction between the hemostatic system and *Candida* infections, especially in neonates with candidemia, remain limited. Despite the high morbidity and mortality due to candidemia, there are insufficient reports evaluating the hemostatic profile of these patients. This lack of information creates a void in the understanding of the way that coagulopathies may affect the clinical outcome of neonates with SCI. There is a need for further research to elucidate the mechanisms governing these interactions and to assess their significance for the clinical management of neonates affected by this dangerous infection. Understanding these mechanisms may contribute to the development of more effective prevention and treatment strategies. The aim of this study was to evaluate the hemostatic profile of neonates with candidemia using the rotational thromboelastometry (ROTEM) method. This study aimed to compare the ROTEM parameter values obtained from neonates with SCI to the corresponding values from healthy neonates. ROTEM is a viscoelastic method that allows real-time assessment of blood coagulation dynamics at the patient’s bedside, providing comprehensive information on the hemostasis system, from the initiation of clot formation to fibrinolysis, and includes both cellular and plasma components [22,23,24,25,26,27,28,29,30]. 

## 2. Materials and Methods

This was a single-center, prospective observational cohort study conducted over a four-year period (April 2020–April/2024) in the neonatal intensive care unit (NICU) at Nikea General Hospital “Agios Panteleimon” in Piraeus, Greece. This study was approved by the Institutional Review Board (approval number: 26-02-20, protocol 5/24) and adhered to the principles outlined in the Declaration of Helsinki. Informed consent was obtained from the parents or guardians of all participants.

### 2.1. Participants

This study involved neonates diagnosed with SCI, and a group of healthy neonates. The criteria for the healthy neonates recruited into the control group had been previously described [22]. Neonates with congenital malformations, incomplete demographic, clinical, or laboratory data, or those who had received fresh frozen plasma, platelets, or anticoagulant therapy prior to performing ROTEM analysis were excluded from this study.

The diagnosis of SCI was confirmed by at least one positive blood, CSF, or a suprapubic aspirate culture for *Candida* species [31]. The onset of the disease was defined as the first occurrence of clinical signs or symptoms such as temperature instability, feeding difficulties, abdominal distension, apnea, dyspnea, retractions, or the need for oxygen or mechanical support, hemodynamic instability, cardiovascular signs such as bradycardia, tachycardia, poor peripheral perfusion, or hypotension, neurological symptoms such as seizures, irritability, lethargy, or hypotonia, and other signs like hyperglycemia, jaundice, petechiae or pallor. The VITEK 2 system (BioMeriéux, Marcy l’Etoile, France), a fully automated platform designed for species identification and antifungal susceptibility testing, was utilized for the identification of *Candida* species.

### 2.2. Study Design

Data were collected on patient demographics, gestational age, birth weight, sex, mode of delivery, maternal and pregnancy history. Laboratory data at the onset of the disease were recorded, including blood culture results (ideally two blood samples before antibiotic initiation), routine biochemical tests (electrolytes, transaminases, urea, creatinine, bilirubin, glucose, and calcium), complete blood count, peripheral blood smear, and C-reactive protein (CRP) levels.

ROTEM assays measure clotting dynamics through the extrinsic (EXTEM) and intrinsic (INTEM) coagulation pathways and assessment of fibrin polymerization (FIBTEM) were performed at disease onset, following the manufacturer’s instructions (Tem Innovations GmbH, Munich, Germany). The following ROTEM parameters were evaluated: clotting time (CT, in seconds), clot formation time (CFT, in seconds), the amplitude recorded at 10 min (A10, in millimeters), maximum clot firmness (MCF, in millimeters), and the lysis index at 60 min (LI60, in percentage) [32].

Neonatal severity scores, including the Modified Neonatal Multiple Organ Dysfunction (NEOMOD) score, and the Neonatal Sequential Organ Failure Assessment (nSOFA) score, were calculated at the onset of the disease [33,34]. Clinical bleeding events were assessed using the Neonatal Bleeding Assessment Tool (NeoBAT) on the same day as ROTEM analysis was performed [35]. Brain and abdominal ultrasounds were conducted weekly and whenever there was suspicion of bleeding or thrombosis, according to NICU protocol.

Fluconazole prophylaxis was administered at a dose of 3 mg/kg, given twice weekly. This regimen was followed for infants born before 28 weeks of gestation, those with birth weight below 1000 g, and/or neonates with additional risk factors, including a history of abdominal surgery, necrotizing enterocolitis (NEC), exposure to broad-spectrum antibacterial agents, or long-term parenteral nutrition, in accordance with the unit’s protocol. Only one neonate was on Fluconazole prophylaxis at the time of enrollment in this study.

### 2.3. Statistical Analysis

Statistical analysis for the study population included descriptive statistics of the data. Means ± standard deviations (SD), medians and interquartile ranges (IQR) were calculated for continuous variables, while frequencies with percentages were calculated for categorical variables. Several parameters including demographics and ROTEM measurements were compared between neonates with SCI and control group using the non-parametric Wilcoxon rank-sum test for continuous variables and the chi square test for categorical variables. The correlation between ROTEM parameters and clinical severity scores was evaluated using the Spearman rank correlation coefficient (Spearman’s rho categories of correlation: r < 0.20, very weak correlation; 0.21 < r < 0.40 weak correlation; 0.41 < r < 0.60, moderate correlation; 0.61 < r < 0.80, strong correlation; r > 0.81 very strong correlation). Last, in order to assess whether SCI is independently associated with altered coagulation dynamics, as reflected by ROTEM parameters, a multivariable linear regression analysis was performed with ROTEM parameters as the dependent parameters, and presence of SCI, sex, birth weight, and gestational age as the independent variables. The Stata 15.0 software (Stata Corp., College Station, TX, USA) was used for the statistical analysis, while statistical significance was set at *p* value < 0.05.

## 3. Results

### 3.1. Baseline Characteristics of Study Cohort

In total, 21 neonates with SCI were included in this study, while the control group consisted of 24 healthy neonates. The most common isolated *Candida* species were *Candida albicans* in 15 (71.42%) cases and *Candida parapsilosis* in 6 (28.58%) cases. Neonates with *Candida* infections and healthy neonates had similar gestational age (*p* = 0.98), while also these two groups did not differ in terms of birth weight (*p* = 0.27), sex (*p* = 0.52), and delivery mode (*p* = 0.17; Table 1). 

Regarding comorbidities, the incidence of respiratory distress syndrome and of intra-ventricular hemorrhage was similar in neonates with SCI and healthy neonates (*p* = 0.51 and *p* = 0.11, respectively; Table 2). Septic shock was evident in nine (42.8%) neonates with SCI, while four (19%) neonates with SCI did not survive. The median modified NEOMOD score for the infected neonates was 6 (IQR: 3–8), while their median nSOFA score was 3.0 (IQR: 2.0–6.0). Last, based on the NEOBAT score, two (9.52%) neonates with SCI experienced severe bleeding events (NEOBAT score ≥ 3).

In terms of the conventional laboratory findings, the neonates with SCI had lower platelet count (medians: 59.0 × 10^3^/mL vs. 293.5 × 10^3^/mL, *p* < 0.001) and lower hematocrit level (medians: 35.2% vs. 45.5%, *p* < 0.001) compared to healthy neonates. The median CRP level in the neonates with SCI was 66.4 mg/L (IQR: 13.1–117.5), while their median WBC was 11570/mL (IQR: 9120–16,140) and their median neutrophile percentage was 50.0% (IQR: 35.8–64.0). 

### 3.2. ROTEM Parameters

Several ROTEM parameters differed between neonates with *Candida* infection and healthy neonates, indicating that these infections may be associated with altered hemostatic dynamics, and a reduced coagulation potential (Table 3). 

Specifically, neonates with SCI had reduced EXTEM A10 (*p* < 0.001) and EXTEM MCF (*p* = 0.003) compared to healthy neonates, while they also had prolonged EXTEM CT (*p* < 0.001) and EXTEM CFT (*p* < 0.001) compared to healthy neonates. Moreover, the diminished coagulation potential in neonates with SCI was also supported by the results of the INTEM method of ROTEM analysis, since neonates with SCI had lower INTEM A10 (*p* = 0.003) and lower INTEM MCF (*p* = 0.003) compared to healthy neonates, while they had prolonged INTEM CFT (*p* = 0.001).

Several ROTEM parameters were also correlated with neonatal clinical severity scores (Table 4). 

Specifically, a strong positive correlation was revealed between EXTEM CFT and the modified NEOMOD score, the nSOFA score and the NeoBAT score. Moreover, a strong negative correlation was evident between EXTEM A10 and the modified NEOMOD score, the nSOFA score and the NeoBAT score. Similar to EXTEM A10, a strong negative correlation was evident between EXTEM MCF and the modified NEOMOD score, the nSOFA score and the NeoBAT score. Similar correlations were evident between INTEM ROTEM parameters and these clinical scores.

The strong association between *Candida* infection in neonates and a diminished coagulation potential, as reflected by a reduced clot amplitude at 10 min and maximum clot firmness, and increased clotting times was also supported by the results of the multivariable linear regression analysis (Table 5).

Specifically, the multivariable regression analysis adjusted for sex, gestational age, and birth weight revealed that *Candida* infection was associated with reduced EXTEM A10, reduced EXTEM MCF, prolonged EXTEM CT, and increased EXTEM CFT. The same INTEM parameters were associated with *Candida* infections as shown in Table 5.

## 4. Discussion

This study evaluated the value and role of ROTEM variables in the early detection of hemostatic disorders in neonates with SCI. In the initial stages of sepsis in the neonates in our study, deviations were observed in the ROTEM parameter values from the standard ROTEM values in healthy neonates, indicating that a hypocoagulable profile is a common early finding among neonates with SCI. 

*Candida* infection is the third most common cause of late-onset sepsis in the NICU, associated with high mortality and hospital costs. *Candida albicans* and *Candida parapsilosis* are the most frequently isolated fungal species in neonatal SCI [36,37]; however, in recent years, reports of systemic infections caused by other *Candida* species, such as *C. auris*, have increased in neonatal patients hospitalized in NICUs worldwide. These outbreaks are associated with poor prognosis, and despite antifungal therapy, infection by *Candida* spp. is often fatal in premature neonates [38,39,40,41]. 

The incidence of SCI in NICUs has increased in recent years due to advances in medical care. Multiple factors contribute to this increase, including the improved survival of extremely premature neonates at the limits of viability, invasive procedures such as central venous and arterial catheterization, gastrointestinal surgeries, and the use of broad-spectrum antimicrobial therapies [38]. These infections also complicate the clinical course of full-term neonates admitted to NICUs with serious underlying health conditions [38,42,43]. There are various international guidelines for the management, therapy and prevention of SCI in neonatal patients with a focus on providing recommendations based on what is known about neonatal infections caused by *C. albicans* and *C. parapsilosis* [44,45,46]. 

Understanding modifiable risk factors for invasive candidiasis, pharmacological prophylaxis, and taking preventive measures have contributed to a decrease in the incidence of candidiasis in high-income countries since the 1990s [12,47]. Although multicenter data from low-income countries are scarce, preventive measures have been shown to reduce the incidence of SCI in these regions as well. However, immune responses, clinical progression, and outcomes of SCI, combined with antifungal prescribing guidelines, have been shown to vary between different *Candida* species [48,49,50,51]. The outcome of SCI depends on the type of *Candida*, possibly because different species of the *Candida genus* exhibit different patterns of virulence factors [52], antifungal sensitivity [53], and differential-species-dependent recognition of the immune system [54]. Therefore, there is a clinical need to comprehend the pathogenesis infection during the neonatal period and to characterize the interactions between *Candida* species and neonates.

The manifestation and severity of candidiasis in the neonatal population are likely due partially to the immaturity and regulation of the immune system in the developing fetus, which matures after birth. Neonatal candidiasis, therefore, represents a unique host–pathogen interface in which both the virulence mechanisms of the fungus and the neonate’s response to infection may differ significantly from those observed in *Candida* infections in other age groups [55]. Mechanisms that are implicated in the recognition, signaling, recruitment and effector response against SCI have been identified and discussed in the international literature. These mechanisms include the presence of antimicrobial peptides, phagocytosis, the production of reactive forms of oxygen, inflammatory mediators, and complex cellular signaling systems through Pattern Recognition Receptors (PRRs) [55,56,57,58]. The complement system, with its multidimensional functions, is involved in immune defense against fungi, and recently several new aspects have emerged in this long-standing battle. It has become clear that *Candida* can adopt both roles, either as a colonist or as a pathogen. Recent findings indicate that *Candida* activates the complement system not only via the classical, lectin, or alternative pathways. An increasing number of indirect mechanisms of complement activation by *Candida* species have been discovered, involving molecules from the contact coagulation system and the fibrinolysis system [59].

Neonatal SCI may initially present with minor or non-specific symptoms that are often attributed to bacterial infections or non-infectious situations. The prognosis and outcome of septic neonates worsen when disseminated intravascular coagulation (DIC) or multiorgan failure develops. For this reason, the existence of diagnostic criteria for the early diagnosis of neonatal *Candida* sepsis is of utmost importance. Activation of the coagulation system is often observed in critically ill patients following sepsis. In sepsis, there is a disruption of the normal delicate balance between prothrombotic and antithrombotic factors, resulting in a severe disturbance of coagulation homeostasis. This leads to systemic thrombin production, reduced anticoagulant activity, and suppression of fibrinolysis. This condition is known as Sepsis-Induced Coagulopathy (SIC) [60,61]. The activation of coagulation resulting from sepsis is accompanied by impaired function of the main anticoagulant mechanisms, including antithrombin, the tissue factor pathway inhibitor system, and fibrinolysis. These disorders lead to the formation of fibrin clots in the microcirculation and tissue hypoxia in organs, while the rapid depletion of coagulation factors simultaneously results in hemorrhages [62,63,64]. Therefore, the close relationship between hemostatic disorders and the inflammatory response of the affected individual constitutes an important parameter in the pathogenesis of a severe infection [65]. Several findings from studies indicate that fungal proteinases are involved in blood coagulation, directly activating coagulation factors, with or without the involvement of complement or antigen–antibody complexes [66]. The limited proteolysis of blood coagulation cascade proteins, specifically the zymogens of serine proteinases, which leads to the activation of these components and is linked to the action of microbial proteinases, may be responsible for hemostatic disorders associated with sepsis, inadequate peripheral circulation and tissue perfusion, DIC, and consequently, multi-organ failure. Thus, fungal proteinases play a significant role in the manifestation of SIC and DIC. The consumption and depletion of endogenous hemostatic factors frequently occur in patients with severe sepsis, as demonstrated in many studies. This disorder may occur even before the clinical diagnosis of sepsis or the onset of acute organ dysfunction directly associated with sepsis [67]. Therefore, the timely diagnosis of hemostatic disorders is a cornerstone in the clinical management of these patients. Conventional coagulation tests, such as prothrombin time and activated partial thromboplastin time, are unable to provide any information or indication regarding platelet functionality or the fibrinolytic system. Additionally, they have limitations in predicting bleeding and guiding transfusion therapy in critically ill patients [28,30]. Viscoelastic tests, such as thromboelastography and ROTEM, allow for the rapid detection of coagulation disorders, providing general information about the dynamics of clot formation and dissolution. Moreover, they serve as ideal tools for monitoring the stages of coagulation, thereby guiding therapeutic intervention with precision [29,30,68,69].

In the present study, it was found that *Candida* species-induced sepsis in neonates causes a hypocoagulable hemostatic response, characterized by prolonged CT (EXTEM), prolonged CFT (EXTEM and INTEM), and decreased values of ROTEM A10 and MCF in the EXTEM and INTEM tests. These findings remained consistent even after adjusting for confounding factors such as gestational age, birth weight, and sex, which have been shown in previous studies to be related to the hemostatic profile of neonates [22,70,71,72,73,74,75]. This finding aligns with the pathophysiology of “consumption of coagulation factors- excessive activation of the coagulation mechanism” during DIC, which is commonly described in adults with sepsis [62,63,64,76].

It should be noted that the ROTEM parameters A10 and MCF incorporate the contribution of platelet count and functionality, fibrinogen concentration, fibrin polymerization, and the role of factor XIII in clot stability [77,78]. Regarding platelets, this study showed that platelet count was significantly reduced in neonates suffering from SCI. Studies have shown that thrombocytopenia is statistically significant in fungal sepsis compared to bacterial sepsis [79]. Τhe study from James M et al. [80] observed that thrombocytopenia was present in 70% of infants with fungal sepsis. Claveras TS et al. [81] concluded that thrombocytopenia is a highly specific marker of neonatal sepsis caused by *Candida*. The study by Parvez A et al. [82] showed that severe thrombocytopenia and a more significant drop in platelet count were observed to a greater extent in fungal sepsis than in bacterial sepsis. Guida JD et al. [83] found a significantly low platelet count and an increase in mean platelet volume at the onset of sepsis in cases of Gram-negative and fungal sepsis. A recent study from Yang & Mao [84] found that monitoring platelet count can be used to track preterm neonates at risk for nosocomial fungal infections. The authors emphasize that fungal infection should be suspected when the platelet count falls below 157 × 10^9^/L. Platelet count has the potential to serve as a convenient and cost-effective marker for the early diagnosis of fungal infections in preterm neonates.

Beyond their primary role in coagulation, platelets actively participate in host defense, being capable of phagocytosing pathogens and producing cytotoxic free radicals and oxidative molecules. It is hypothesized that a combination of factors, such as diffuse endothelial cell damage, bacterial or fungal toxins, increased platelet activation, and disseminated DIC, leads to heightened platelet consumption during sepsis [85]. Studies have shown that VLBW neonates exhibit a limited ability to respond to thrombocytopenia, both in platelet production and in thrombopoietin secretion [86]. This response may be further impaired during sepsis episodes, where the host has reduced energy reserves and may have sustained liver damage, limiting the ability to replenish platelets [87].

Given that invasive fungal infections are associated with significant morbidity and mortality, with reported 30-day mortality rates as high as 43%, researchers and clinicians have evaluated the risk factors that lead to early diagnosis and intervention [88]. For example, it has been found that patients who died from invasive fungal infections had very low platelet counts and RANTES (regulated on activation, normal T-cell expressed and secreted) concentrations, suggesting that these factors may play a role in the host’s response to such infections [89]. This is expected, as platelets are the primary source of circulating RANTES, and platelet count is significantly correlated with RANTES levels. Additionally, platelet microparticles (PMPs), small particles released from activated platelets that express CD42a and GPIIbIIIa, have been found to increase significantly in patients with fungal sepsis compared to the control group of non-fungal sepsis [90]. Specifically, in liver transplant recipients, patients with thrombocytopenia were at increased risk of developing fungal infections [91]. Aside from the clinical evidence suggesting platelet products as markers of fungal disease, in the case of Immune Thrombocytopenic Purpura (ITP), fungal disease emerged as a significant prognostic factor for the diagnosis of ITP [92]. *C. albicans* does not appear to affect the release of alpha or dense granules, but it significantly weakens platelet aggregation in response to multiple agonists. Additionally, platelets can directly kill *C. albicans* through the release of their granular contents, while *C. albicans* can also exert inhibitory effects on platelet aggregation [21]. It is worth noting that in our study, no significant differences were observed in the FIBTEM parameters of ROTEM after adjustment for confounding factors, which may likely be attributed to the inhibition of platelet participation in the clot formation process due to cytochalasin D. This finding, along with the low clot size in the EXTEM and INTEM ROTEM tests (A10, MCF), supports the theory that platelets play a significant role in the pathophysiology of neonatal *Candida* infection.

The human plasma contact system represents a powerful tool for immune surveillance, which is activated by negatively charged surfaces (e.g., on fungi). The contact of factor XII (FXII) with these surfaces leads to its autoactivation and conversion into the active serine protease FXIIa. This process is also triggered by contact with the cell walls of *Candida albicans* and *C. tropicalis* [93]. Additionally, previous studies have shown that proteases derived from *Candida* activate factor XII, which appears to be a multifunctional molecule in innate immunity [94,95]. The active form of FXII, whether derived from surface contact with *Candida* or from *Candida* proteases, separates prekallikrein to form kallikrein. Kallikrein further activates FXII, rapidly increasing the plasma levels of both of these enzymes [95,96]. Additionally, studies have shown that proteases from *Candida albicans* can directly convert plasma prekallikrein into active kallikrein. The critical role of proteases from *Candida, Porphyromonas gingivalis*, and other proteases in enhancing vascular permeability has also been reported, a crucial inflammatory process induced by bradykinin through the activation of factor XII, prekallikrein, or both [94,97].

Activated FXII, whether from *Candida* surfaces or *Candida* proteases, also plays a central role and promotes the formation of active thrombin, a key enzyme in coagulation [98]. Active thrombin can also be generated directly through the enzymatic action of *Candida* proteases [99]. It has been reported that *Candida* proteases activate either factor XII or prothrombin in vitro, leading to the generation of thrombin. Factor X is also converted into its active form (Xa) by *Candida* proteases [94]. These findings suggest that both peripheral and systemic blood circulation can be affected by the activation of the coagulation cascade, not only by microbial infections but also in cases of *Candida* infection, particularly in systemic infections. All of the aforementioned are confirmed by the findings of our study, where, in the ROTEM variables of the INTEM test, which evaluates the intrinsic coagulation pathway, a hypocoagulable profile was observed in neonates with *Candida* infection. This profile remained consistent even after adjustment for confounding factors such as gestational age, birth weight, and sex. 

Studies in adults have reported an interaction between inflammation and hemostasis, primarily through three mechanisms: elevated levels of tissue factor (TF), suppression of the anticoagulant pathway, and fibrinolysis disturbances. TF is the main trigger for coagulation activation in inflammation. The increased production of TF is attributed both to endothelial exposure to circulation after vascular injury and to its presence in immune system cells [29] The activity of tissue factor in hematopoietic cells has been found to significantly change in the presence of *C. albicans* [100]. The results of our study, where the affected neonates exhibited a hypocoagulable profile according to the ROTEM parameters in the EXTEM test, may support the hypothesis of involvement of the extrinsic coagulation pathway in systemic *Candida* infection.

In our study, several ROTEM parameters were also correlated with clinical severity scores. Specifically, a strong positive correlation was revealed between EXTEM-INTEM CFT and the modified NEOMOD score, the nSOFA score, and the NeoBAT score. Additionally, a strong negative correlation was evident between EXTEM-INTEM A10, EXTEM MCF and the modified NEOMOD score, as well as between EXTEM-INTEM A10, EXTEM-INTEM MCF, and the nSOFA score and the NeoBAT score. This hypocoagulable profile reflects the hemostatic disturbances, which seem to contribute to both the poor prognosis and the increased risk of hemorrhagic complications in this population. In our study, 19% of neonates with *Candida* infection exhibited intraventricular hemorrhage (IVH) grade I, and 14.3% had severe IVH grade ≥ III (according to the criteria of Papile et al. [101]), and 9.52% of neonates with SCI experienced severe bleeding events (NEOBAT score ≥ 3). Similarly, Adalarasan [102] and colleagues conducted a study where they assessed the relationship between *Candida* infection and the occurrence of intracranial hemorrhage (ICH) and intraventricular hemorrhage (IVH) in 80 neonates. Of these, 9 had ICH, 21 had IVH, and 50 did not show any bleeding. The findings suggest that even term neonates are not exempt from complications associated with fungal sepsis, highlighting the severity of fungal infections as a risk factor for hemorrhagic complications in neonates, regardless of gestational age. Additionally, previous studies from our research group have shown that the values of ROTEM variables such as CT, CFT, MCF, A10, and A20 reflect not only the degree of hemostatic insufficiency in septic neonates with prominent bleeding tendency but also their outcomes [24,32,103,104], which aligns with the findings of studies involving adult patients with sepsis [105,106].

Studies report an association between antifungal drugs and hemostatic disorders, with echinocandins showing a higher correlation than triazoles and polyenes. Specifically, of the three echinocandins, anidulafungin showed the strongest association with coagulation dysfunction, followed by micafungin and caspofungin [107]. However, although the exact causality of this relationship requires confirmation through further randomized controlled trials, these findings, combined with the hypocoagulable profile of neonates with *Candida* infection observed in our study, highlight the importance of evaluating the hemostatic profile of these neonates. This underscores the need for a personalized medical approach, as well as the quantification of risk and the assessment of benefit for each neonate before deciding on the appropriate therapeutic strategy. In this way, it can be ensured that the intervention will be targeted and effective, minimizing potential complications.

This study has certain limitations, as it is single-center and involves a small patient sample, necessitating careful interpretation of the results. The correlation between the ROTEM parameters and conventional coagulation tests was not run due to the lack of available data. Additionally, the variation in measurement time and the absence of serial measurements may have influenced the results, as *Candida* sepsis is a dynamic process. Coagulation assessment is complex and requires serial measurements, which were absent from this study, limiting the evaluation of therapeutic response. On the other hand, the fact that the data come from a single center reduces the impact of different clinical practices, ensuring homogeneity in the recognition of early clinical signs and symptoms of *Candida* infection at the onset of the disease.

## 5. Conclusions

This study highlighted the importance of ROTEM parameters in the early detection of hemostatic disorders in neonates with systemic *Candida* infection. The hypocoagulable profile, as expressed through significant prolongation of CT and CFT times, as well as reduced clot strength (A10 and MCF), is a common early finding in this population. The correlations of these findings with clinical severity scores (modified NEOMOD, nSOFA, NeoBAT) emphasize the crucial role of hemostatic disturbances in poor outcomes and the increased risk of hemorrhagic complications in neonates with *Candida* infection. This research may contribute to the recognition and understanding of hemostatic derangement that can occur in neonates with candidemia, providing valuable data to improve clinical care and prognosis for these patients. However, further studies with a larger sample size are required to confirm the findings and assess their contribution to the timely and accurate management of neonates, who are already a high-risk group.

## Figures and Tables

**Table 1:** Baseline characteristics of the study cohort.

	Neonates with *Candida* Infection (*n* = 21)	Control Group (*n* = 24)	*p*-Value
Gestational age (weeks)	34.1 ± 4.7; 34.0 (29.0–40.0)	34.0 ± 4.5; 34.0 (29.5–40.0)	0.98
Birth weight (g)	2276.5 ± 1178.8, 3300.0 (1145.0–3300.0)	2380.0 ± 1020.0; 2212.5 (1420.0–3335.0)	0.27
Sex (males, %)	13 (61.9)	17 (70.8)	0.58
Delivery mode (Caesarean section, %)	17 (80.9)	15 (62.5)	0.17

Data are presented as means ± SD, medians and interquartile range (IQR), or as frequencies (percentages) when appropriate. The Chi-square and the two-sample Wilcoxon rank-sum (Mann–Whitney) test were used for comparison between the groups.

**Table 2:** Clinical characteristics of the study population.

	Neonates with *Candida* Infection (*n* = 21)	Control Group (*n* = 24)	*p*-Value
IUGR (%)	4 (19.0)	0 (00.0)	0.025
RDS (%)	14 (66.6)	9 (37.5)	0.051
Septic shock	9 (42.8)	-	-
Acute renal failure (%)	8 (38.0)	0 (00.0)	0.001
IVH (%)	7 (33.3)	3 (12.5)	0.11
Grade I	4 (19.0)	3 (12.5)	
Grade III	3 (14.3)	0 (00.0)	
Deaths (%)	4 (19.0)	0 (00.0)	**-**
Modified NEOMOD score	6.3± 3.3, 6.0 (3.0–8.0)	-	**-**
NeoBAT score (%)		-	-
Grade 1	5		
Grade 2	4		
Grade 3	1		
Grade 4	1		
nSOFA score	3.5± 2.6, 3.0 (2.0–6.0)	-	**-**

*Abbreviations:* IUGR, intrauterine growth restriction; RDS, respiratory distress syndrome; DIC, disseminated intravascular coagulopathy; IVH, intraventricular hemorrhage; NEOMOD, Neonatal Multiple Organ Dysfunction; NeoBAT, Neonatal Bleeding Assessment Tool; nSOFA, Neonatal Sequential Organ Failure Assessment.

**Table 3:** ROTEM parameters among neonates with *Candida* infection and control group.

	Neonates with *Candida* Infection (*n* = 21)	Control Group (*n* = 24)	*p*-Value
EXTEM CT (s)	62.6 ± 14.7, 62.0 (51.0–67.0)	48.7± 11.6, 48.5 (40.5–54.5)	**<0.001**
EXTEM CFT (s)	306.2 ± 362.1, 182.0 (104.0–345.0)	85.7 ± 82.5, 82.5 (60.0–98.5)	**<0.001**
EXTEM A10 (mm)	37.7 ± 14.7, 35.0 (25.0–47.0)	55.1 ± 9.0, 55.5 (50.0–60.5)	**<0.001**
EXTEM MCF (mm)	48.2 ± 16.7, 48.0 (33.0–64.0)	65.4 ± 14.0, 61.5 (56.0–72.0)	**0.003**
EXTEM LI60 (%)	97.1 ± 3.3, 97.5 (96.0–100.0)	92.7 ± 11.6, 96.5 (93.0–98.0)	0.12
INTEM CT (s)	251.2± 127.1, 219.0 (196.0–246.0)	194.8± 29.5, 202.0 (177.0–218.0)	0.21
INTEM CFT (s)	376.1± 455.1, 117.5 (99.0–587.5)	82.8± 33.9, 74.0 (62.0–103.0)	**0.001**
INTEM A10 (mm)	37.0± 16.1, 42.5 (21.5–50.5)	53.0± 9.1, 53.0 (45.0–60.0)	**0.003**
INTEM MCF (mm)	49.9± 18.3, 55.0 (33.0–60.5)	58.8± 6.5, 61.0 (53.0–63.0)	**0.003**
INTEM LI60 (%)	95.7± 10.9, 98.5 (97.0–100.0)	93.8± 2.9, 94.0 (92.0–96.0)	**<0.001**
FIBTEM CT (s)	53.5 ± 14.2, 55.0 (47.0–62.0)	48.8± 7.0, 47.0 (43.0–56.0)	0.09
FIBTEM A10 (mm)	20.3 ± 7.3, 19.5 (15.0–24.0)	16.1 ± 5.1, 16.0 (14.0–19.0)	0.12
FIBTEM MCF (mm)	32.9 ± 22.1, 25.0 (19.0–37.0)	-	-
FIBTEM LI60 (%)	93.2 ± 24.9, 100.0 (100.0–100.0)	98.6 ± 2.3, 100.0 (99.0–100.0)	0.28

*Abbreviations:* CT, clotting time; CFT, clot formation time; A10, clot amplitude at 10 min; MCF, maximum clot firmness; LI60, lysis index at 60 min; data are presented.

**Table 4:** Correlation of ROTEM parameters with modified NEOMOD, nSOFA and NeoBAT scores.

Variables	Modified NEOMOD		nSOFA		NeoBAT	
	Spearman’s rho	*p*-Value	Spearman’s rho	*p*-Value	Spearman’s rho	*p*-Value
EXTEM CT	0.36	0.10	0.45	**0.036**	0.45	**0.037**
EXTEM CFT	0.76	**<0.001**	0.66	**<0.001**	0.70	**<0.001**
EXTEM A10	−0.76	**<0.001**	−0.71	**<0.001**	−0.72	**<0.001**
EXTEM MCF	−0.71	**<0.001**	−0.74	**<0.001**	−0.63	**0.001**
EXTEM LI60	0.30	0.25	0.01	0.96	0.23	0.37
INTEM CT	0.21	0.40	−0.008	0.97	−0.02	0.92
INTEM CFT	0.75	**<0.001**	0.77	**<0.001**	0.74	**<0.001**
INTEM A10	−0.60	**0.013**	−0.75	**<0.001**	−0.66	**0.005**
INTEM MCF	−0.48	0.056	−0.68	**0.003**	−0.62	**0.010**
INTEM LI60	0.54	**0.044**	0.40	0.15	0.43	0.11
FIBTEM CT	−0.07	0.76	0.15	0.53	0.02	0.93
FIBTEM A10	−0.27	0.27	−0.49	**0.036**	−0.17	0.49
FIBTEM MCF	−0.26	0.26	−0.44	0.057	0.02	0.91
FIBTEM LI60	−0.20	0.43	0.14	0.60	−0.11	0.66

*Abbreviations:* NEOMOD, Neonatal Multiple Organ Dysfunction; nSOFA, Neonatal Sequential Organ Failure; NeoBAT, Neonatal Bleeding Assessment Tool; CT, clotting time; CFT, clot formation time; A10, clot amplitude at 10 min; MCF, maximum clot firmness; LI 60, lysis index at 60 min.

**Table 5:** Results of multivariable regression analysis for ROTEM parameters as dependent variables, with gestational age, birth weight, sex, and presence of *Candida* infection as independent variables.

ROTEM Parameters	*Candida* Infection		
	Coefficient	95% CI	*p*-Value
EXTEM CT (s)	13.4	5.2–21.5	**0.** **002**
EXTEM CFT (s)	211.8	74.7–348.8	**0.0** **03**
EXTEM A10 (mm)	−17.3	−23.8–−10.9	**<** **0.00** **1**
EXTEM MCF (mm)	−15.9	−23.6–−8.3	**<0** **.001**
EXTEM LI60 (%)	5.0	−1.3–11.3	0.11
INTEM CT (s)	53.9	−16.0–123.8	0.12
INTEM CFT (s)	361.7	140.9–582.5	**0.** **002**
INTEM A10 (mm)	−23.3	−33.3–13.3	**<** **0.00** **1**
INTEM MCF (mm)	−13.3	−21.8–−4.8	**0.0** **03**
INTEM LI60 (%)	2.9	−2.7–8.7	0.29
FIBTEM CT (s)	3.0	−5.9–12.0	0.49
FIBTEM A10 (mm)	5.0	1.4–8.5	**0.** **006**
FIBTEM MCF (mm)	4.3	−0.8–9.5	0.09
FIBTEM LI60 (%)	−10.3	−24.8–4.1	0.15

*Abbreviations:* CI, confidence interval; CT, clotting time; CFT, clot formation time; A10, clot amplitude at 10 min; MCF, maximum clot firmness; LI60, lysis index at 60 min.

## Data Availability

The original contributions presented in this study are included in the article; further inquiries can be directed to the corresponding authors.
